# Nursing diagnoses among oncology patients in medical units: a retrospective study of patients’ records

**DOI:** 10.3332/ecancer.2021.1315

**Published:** 2021-11-04

**Authors:** Elham H Othman, Mohammad R Alosta, Jafar Alasad Alshraideh, Shahd Al Muhaisen

**Affiliations:** 1 School of Nursing, Applied Science Private University, Amman, 11931, Jordan; 2 School of Nursing, Zarqa University, Zarqa, 13110, Jordan; 3 School of Nursing, University of Jordan, Amman, 11942, Jordan; 4 School of Medicine, University of Jordan, Amman, 11942, Jordan; a https://orcid.org/0000-0002-2438-0429; b https://orcid.org/0000-0003-1710-3391; c https://orcid.org/0000-0002-2118-5257; d https://orcid.org/0000-0003-1566-7660

**Keywords:** classification, documentation, nursing diagnosis, oncology nursing, patient care planning, standardised nursing terminology

## Abstract

**Objective:**

Nursing care plans for oncology patients are complex and overlapping enough to warrant the need for systematised documentation that ensures high quality, flawless and comprehensive care. Addressing the patients’ needs through nursing diagnoses is the initial step that shapes the subsequent care. Therefore, the current study aimed to identify the frequent NANDA-I diagnoses reported in nursing care plans for medical oncology patients.

**Data sources:**

A retrospective design was used to collect data from 260 electronic nursing care records of oncology patients admitted to medical floors at an accredited oncology centre in Jordan.

**Conclusion:**

The complexity of nursing care for oncology patients can be inferred from the high number of reported nursing diagnoses. This study summarises the most common nursing diagnoses and their combinations that can be used as a guide to formulate nursing care plans for oncology patients in medical units.

**Implications for nursing practice:**

Oncology nurses may refer to this study to guide and support their care and documentations to maintain a high standard of nursing practice. Besides, the reported diagnoses can be integrated to generate pre-printed, standardised nursing care plans, where diagnoses are listed for nurses to select the applicable ones for their patients. Similarly, the combinations of nursing diagnoses may guide nurses to search for a concurrent diagnosis, thus improving patients’ outcomes. This study revealed the complexity of patients’ care in medical oncology units, which alarms the nursing managers to reconsider the nurse–patient ratio in these settings to meet patients’ care demands and maintain their safety.

## Introduction

### Background

With an estimated 9.6 million deaths in 2018, cancer is the second leading cause of mortality [1]. Providing care for patients with cancer is complex due to the nature of the disease. Nurses provide comprehensive care for cancer patients based on knowledge, technical-scientific skills and interpersonal skills [2]. The nursing process provides a systematic tool for delivering the nursing care plan across all settings in which professional nursing care takes place. Accurately written nursing care plans usually distinguish nurses’ contributions to patients’ outcomes and recognise the nursing profession.

The standardised nursing terminology is used to plan the nursing process, guide nursing actions and documentation and improve communications about patients’ health [3]. The development of standardised nursing classification systems began in the 1970s [4]. These classification systems are classified into three categories: (1) nursing diagnoses or problems, such as the NANDA-I; (2) nursing interventions or actions, such as the nursing interventions classification (NIC) and (3) nursing outcomes or assessment, such as the nursing outcomes classification (NOC) [5].

The reasons for patients’ admissions determine nursing diagnoses. Moreover, the number of nursing diagnoses per patient can be used to predict hospital length of stay [6, 7]. Nursing interventions (derived from the NIC) are central for caring for patients with cancer [8]. Similarly, the NOC is used to evaluate the patient’s response to the interventions provided.

The NANDA-NOC-NIC linkage can collectively contribute to support the nursing process, care plans applications and encourages the development of standards in nursing practice for patients with cancer [9, 10].

### Objectives of the study

The purposes of the study were to (1) identify the most frequent NANDA-I nursing diagnoses reported in nursing care plans for oncology patients admitted to medical units and (2) identify patterns of nursing diagnoses combinations among oncology patients admitted to medical units.

## Methods

### Design

A retrospective design was used to collect data from the electronic nursing care plan records at an accredited oncology centre in Jordan.

### Sample and settings

A census method was used to collect data from the records of all patients admitted to the medical floor between January 2019 and October 2019 for medically related problems. Any patients admitted to the floor for non-medical reasons such as pre-procedures/preoperative, replacement of catheter, catheter removal and palliative care were excluded. The study protocol was approved by the Institutional Review Board at the selected hospital. Patients’ records were assigned a pseudo-ID-numbers and data were analysed and reported anonymously.

### Data collection and analysis

Nursing records of eligible patients were retrieved from the admission office in November–December 2019. Two trained nurses assessed all patients’ records and completed the data collection form that consisted of socio-demographic data, medical information and a list of nursing diagnoses and their frequency during hospitalisation. The documented nursing diagnoses were compared to the NANDA-I (2018–2020) list [11]; any documented diagnosis not written according to the NANDA-I was excluded.

Data were coded numerically and analysed using the Statistical Package for Social Sciences (SPSS, Version 21.0). Descriptive statistics were used to describe demographic and medical data. Also, the average number of different diagnoses per patient was calculated. To describe the most frequent NANDA-I nursing diagnoses, the reported nursing diagnoses were categorised according to the NANDA-I domains [11]. Descriptive analyses were performed using frequency distribution and percentages. Additionally, the chi-square test was used to examine any two combinations of the reported nursing diagnosis; the Fisher’s exact test was reported if the expected cell count was less than five. Co-occurrences of two diagnoses were considered significant and reported in the final data analysis results if *p* < 0.05.

## Results

### Sample characteristics

A total of 655 patients were admitted to the medical floor during January–October 2019; of those, 305 patients were admitted for non-medical reasons such as pre-procedures/preoperative, replacement of catheter, catheter removal and palliative care and therefore were excluded from records review. The remaining 260 patients were identified as being admitted for medical problems, and their records were accessed and reviewed to identify the reported nursing diagnoses.

The majority of patients were female (57.7%, *n* = 150). The mean age of the sample was 51.3 years. The participants were classified as young adults (17%, *n* = 44), middle-aged adults (40.7%, *n* = 106) and older adults (42.3%, *n* = 110). Patients were classified according to the type of cancer using the International Classification of Diseases for Oncology, third edition [12]. Patients’ cancer types were: carcinoma (73.1%, *n* = 190), lymphoma (14.6%, *n* = 38), sarcoma (8.5%, *n* = 22) and myeloma (3.8%, *n* = 10). Further, 74 patients (29%) had diabetes mellitus, while 94 (36%) had hypertension. The mean length of hospital stay was 4.5 days (2–16 days). Reasons for admission were categorised as cancer treatment modalities or symptom management. Patients’ characteristics are summarised in Table 1.

### Reported nursing diagnoses

We identified 33 nursing diagnoses; the average diagnoses per patient were 7.25, range (2–16). Three of the reported nursing diagnoses were not following NANDA-I diagnoses and were excluded from the analysis; risk for chemotherapy side effects (*n* = 22), risk of seizure (*n* = 14) and vomiting (*n* = 4). The remaining 30 diagnoses were written according to the NANDA-I under seven domains and 15 classes. The domains were nutrition, elimination and exchange, activity/rest, perception/cognition, safety/protection, comfort and coping/stress tolerance (Table 2).

Cross-tabulation of the reported nursing diagnoses was done to identify patterns of nursing diagnoses combinations, which are clusters of nursing diagnoses that occur together in the same patient; nurses must address these diagnoses to assure holistic patient care. Significant combinations, according to the Chi-square and exact Fisher’s test, are summarised in Table 3.

## Discussion

The study aimed to describe the nursing diagnoses reported in nursing care plans for Jordanian oncology patients in medical units. The nursing diagnosis is considered a tool applied in daily nursing care to facilitate the care process, support nurses’ autonomy and reflect reasons for admission and problems during hospitalisation [13–15]. Reasons for admission in the current study were classified into two main categories: planned admissions for cancer treatments and urgent admissions for symptoms management. Similar percentages were reported in previous studies [6, 16]. Further, an in-depth analysis of reasons for urgent admissions showed that ‘shortness of breath’ was the most commonly reported reason for admission among our patients, which is similar to previous reports by Numico *et al* [16], where the chief complaint was breathlessness.

Most diagnoses in our study were identified as NANDA-I diagnoses, and 3 (9%) diagnoses that were not found in this terminology were excluded. These results showed higher compliance with the NANDA-I nursing diagnoses than a previously published study [6] that analyse nursing records of 150 female patients diagnosed with breast cancer from three Japanese hospitals. The latter study revealed that most nursing diagnoses were not written according to the NANDA-I. The high compliance with NANDA-I in our study supports the results of a previous study within the Jordanian context, where nursing students showed a positive attitude toward using NANDA-I nursing diagnosis [17]. Further, this could be due to the emphasis placed on the concept during the intense nursing education that is continuously provided to nurses by the hospital.

We found that the average nursing diagnoses per patient were 7.25, range from 2 to 16 diagnoses during an average length of stay of 4.45 days. This shows the importance of nursing diagnosis in guiding nurses who care for oncology patients with different admission reasons. These results were consistent with the result of a previous retrospective study [18] that examined nursing diagnoses of 2,237 patients admitted to medical oncology units; they reported an average of 3.1 nursing diagnoses per patient, ranging from 1 to 28 diagnoses during an average length of stay of 3.7 days. Our result also highlights the complexity of patients’ care in medical oncology units, which implies the need to reconsider the nurse–patient ratio in these settings to meet patients’ care demands.

The 30 identified nursing diagnoses comprised 20 problem-focused diagnoses and 10 risk diagnoses. The problem-focused diagnosis is concerned with an existed undesirable human response to a health condition/life process, while the risk diagnosis represents the vulnerability for developing an undesirable human response to health conditions/life processes [19].

The diagnoses were distributed into 15 classes across seven main domains. These domains were reported in a previous review of four research studies [20]. The review identified 40 nursing diagnoses, 34 problem-focused diagnoses and 6 risk diagnoses belonging to 12 domains. In our study, the three most common nursing diagnoses were risk for fall, risk for infection and impaired skin integrity. This can be explained by linking these diagnoses to three nursing quality indicators (falling, hospital-acquired infections and hospital-acquired pressure injury, respectively), regularly reported by the oncology centre where the study was conducted. Hence, nurses may overuse these diagnoses to continuously assess the patients and decrease the incidence of these events.

The combinations of nursing diagnoses in the same patients may help interpret symptoms clusters for the oncology population and guide nurses to look for concurrent nursing diagnoses to provide comprehensive nursing care. The most frequently reported diagnoses combination was chronic pain and impaired skin integrity, as the majority of the patients were found to have both nursing diagnoses concurrently. This is congruent with previous studies that described pain as an issue within the daily life of patients who have pressure injuries [21, 22]. Besides, the clinical impact of this combination of diagnoses would alert nurses to constantly assess the pain among their patients with impaired skin integrity. The second most frequently reported diagnoses combination was altered physical mobility and impaired skin integrity. This finding supports previous studies that reported immobility as a risk factor for pressure injury [23, 24] and included it in the pressure injury risk assessment scales, such as the Braden scale [25].

The current study was conducted in a leading oncology centre in Jordan and the Middle East that recruits professional health care providers from different specialities and provides care for a high number of patients with different cancer diagnoses per year. This is the first study to report the frequency of used nursing diagnoses among oncology patients in Jordan and the region. The study reports nursing diagnosis without referring to nursing interventions and outcomes (the remaining parts in the nursing diagnosis, nursing outcomes, and nursing interventions linkage), as these are not mandated to be documented in patients’ records in the hospital. Further, the results’ generalizability might be affected by the settings, as data were collected from one site only, and that the results might be influenced by nurses’ practice variability and professional training.

### Implications for nursing

Oncology nurses may refer to this study to guide them and support their care and documentations to maintain a high standard of nursing practice. Besides, the reported diagnoses can be integrated to generate pre-printed, standardised nursing care plans, where diagnoses are listed for nurses to select the applicable ones for their patients. Similarly, the combinations of nursing diagnoses may guide nurses to search for a concurrent diagnosis, thus improving patients’ outcomes. Nurse educators and clinical instructors may refer to the identified diagnoses to train students on preparing nursing care plans for medical oncology patients. In terms of leadership implications, this study revealed the complexity of patients’ care in medical oncology units, which alarms the nursing managers to reconsider the nurse–patient ratio in these settings to meet patients’ care demands and maintain their safety.

## Conclusion

Nursing care plans are used to guide nursing care to provide comprehensive, evidence-based practices. The complex nature of oncology patients demands that nurses be updated and systematic in their care and documentation. The current study results provide a summary of NANDA-I nursing diagnoses reported among oncology patients admitted for medical problems. We identified 20 problem-focused diagnoses and 10 risk diagnoses under seven domains and 15 classes. The domains were nutrition, elimination and exchange, activity/rest, perception/cognition, safety/protection, comfort and coping/stress tolerance.

Another significant finding is the reported diagnoses combinations, such as chronic pain and impaired skin integrity; altered physical mobility and impaired skin integrity; chronic pain and impaired physical mobility. Future research studies are needed to address the NIC and NOC’s linked to the identified nursing diagnoses and their impact on patients’ outcomes.

## List of abbreviations

NIC, Nursing Interventions Classification; NOC, Nursing Outcomes Classification; NANDA-I, NANDA-International Inc.

## Conflicts of interest

The authors declare no conflicts of interest.

## Funding

This research received no specific grant from any funding agency in the public, commercial or not-for-profit sectors.

## Figures and Tables

**Table 1.** Reasons for admission to medical floor among oncology patients (*N* = 260).

Reasons for admission	*n*	%
**For cancer treatment** Chemotherapy Radioactive iodine therapy Radiation therapy	96 52 34 10	37% 20% 13% 3.8%
**For symptoms management**	164	63%
***Respiratory system*** SOB Chest infection Pleural effusion Haemoptysis	38 28 6 2 2	14.6% 10.8% 2.3% 0.8% 0.8%
***Digestive system*** Bowel obstruction Vomiting Abdominal pain Decreased oral intake Diarrhoea Ascites Gastrointestinal bleeding Dysphagia	38 8 8 6 4 4 4 2 2	14.6% 3.1% 3.1% 2.3% 1.5% 1.5% 1.5% 0.8% 0.8%
Immune system**** Fever Neutropenic fever	34 24 10	13% 9.2% 3.8%
***Nervous system*** Decreased LOC Pain crisis Seizure Vomiting & headache Agitation Dizziness	20 8 4 2 2 2 2	7.7% 3.1% 1.5% 0.8% 0.8% 0.8% 0.8%
***Circulatory system*** Fatigue Electrolyte imbalances DVT Pancytopenia	18 10 4 2 2	6.9% 3.8% 1.5% 0.8% 0.8%
***Integumentary system*** Cellulitis Infected wound Bleeding skin lesions Abscess	10 4 2 2 2	3.8% 1.6% 0.8% 0.8% 0.8%
***Renal system*** Decreased UOP Haematuria	6 4 2	2.3% 1.5% 0.8%

SOB, Shortness of breath; DVT, Deep vein thrombosis; LOC, Level of consciousness; UOP, Urine output

**Table 2.** Reported nursing diagnosis NANDA-I (N = 260).

Domain	Class	Nursing diagnosis	*n*	%
Nutrition	Ingestion	Imbalanced nutrition: less than body requirements	82	32%
	Hydration	Risk for electrolytes imbalance	68	26%
		Deficient fluid volume	4	1.5%
		Excess fluid volume	2	1%
Elimination and exchange	Gastrointestinal function	Constipation/diarrhoea	42	16%
	Urinary function	Impaired urinary elimination	28	11%
	Respiratory function	Impaired gas exchange	24	9%
Activity/rest	Activity/exercise	Impaired physical mobility	162	62%
	Cardiovascular/pulmonary responses	Ineffective breathing pattern	72	28%
		Activity intolerance	46	18%
		Ineffective peripheral tissue perfusion	24	9%
	Energy balance	Fatigue	18	7%
	Self-care	Self-care deficit	4	1.5%
		Risk for decreased cardiac tissue perfusion	2	1%
Perception/cognition	Cognition	Deficient knowledge	18	7%
		Acute confusion	16	6%
Safety/protection	Infection	Risk for infection	248	95%
	Physical injury	Risk for fall	260	100%
		Impaired skin integrity	224	86%
		Risk for pressure injury	84	32%
		Risk for aspiration	36	14%
		Ineffective airway clearance	20	8%
		Risk for bleeding	16	6%
		Risk for impaired tissue integrity	8	3%
		Risk for impaired skin integrity	6	2%
		Risk for injury	4	1.5%
	Thermoregulation	Hyperthermia	6	2%
Comfort	Physical comfort	Chronic pain	214	82%
		Impaired comfort	6	2%
Coping/stress tolerance	Coping responses	Anxiety	100	38%

**Table 3.** Combinations of reported NANDA-I (*N* = 260).

Nursing diagnoses combination	n	%	p
Chronic pain*Impaired skin integrity	194	75%	0.004^a^
Altered physical mobility*Impaired skin integrity	156	60%	0.001^a^
Chronic pain*Impaired physical mobility	148	57%	0.001^a^
Chronic pain*Risk for electrolytes imbalances	82	31.5%	0.006^a^
Imbalanced nutrition: less than body requirements*Impaired skin integrity	80	30.8%	0.012^a^
Chronic pain*Imbalanced nutrition: less than body requirements	78	30%	0.012^a^
Altered physical mobility*Risk for electrolytes imbalances	70	30%	0.002^a^
Altered physical mobility*Imbalanced nutrition: less than body requirements	68	26.2%	<0.001^a^
Constipation/Diarrhoea*Impaired skin integrity	42	16.2%	0.04^a^
Imbalanced nutrition: less than body requirements*Risk for electrolyte imbalances	40	15.4%	0.015^a^
Imbalanced nutrition: less than body requirements*Risk for pressure injury	38	14.6%	0.027^a^
Ineffective breathing pattern*Risk for electrolytes imbalances	36	13.8%	0.013^a^
Altered physical mobility*Impaired urinary elimination	28	10.8%	0.001^a^
Activity intolerance*Imbalanced nutrition: less than body requirements	26	10%	0.004^a^
Activity intolerance*Risk for electrolyte imbalances	26	10%	0.008^a^
Constipation/Diarrhoea*Imbalanced nutrition: less than body requirements	26	10%	0.002^a^
Imbalanced nutrition: less than body requirements*Impaired urinary elimination	24	9.2%	0.001> ^a^
Ineffective breathing pattern*Risk for aspiration	22	8.5%	0.001>^a^
Ineffective breathing pattern*Ineffective airway clearance	16	6.2%	0.001^a^
Anxiety*Deficient knowledge	16	6.2%	0.002^a^
Risk for electrolytes imbalances*Fatigue	16	6.2%	0.001^a^
Risk for electrolytes imbalances*Ineffective airway clearance	16	6.2%	0.002^a^
Risk for electrolytes imbalances*Impaired tissue perfusion	16	6.2%	0.02^a^
Fatigue*Risk for pressure injury	16	6.2%	0.001>^a^
Ineffective breathing pattern*Impaired gas exchange	12	4.6%	0.001>^a^
Activity intolerance*Fatigue	10	4%	0.009^a^
Confusion*Ineffective breathing pattern	10	3.8%	0.037^a^
Confusion*Risk for aspiration	10	3.8%	0.004^a^
Risk for electrolytes imbalances*Impaired gas exchange	10	3.8%	0.015^a^
Ineffective airway clearance*Risk for aspiration	10	3.8%	0.001>^a^
Activity intolerance*Impaired gas exchange	8	3.1%	0.009^a^
Confusion*Impaired urinary elimination	6	2.3%	0.041^a^
Risk for electrolytes imbalances*Hyperthermia	6	2.3%	0.034^a^
Fatigue*Ineffective airway clearance	6	2.3%	0.022^a^
Hyperthermia*Impaired tissue perfusion	4	1.6%	0.022^b^
Activity intolerance*Deficient fluid volume	4	1.5%	0.03^b^
Excess fluid volume*Impaired gas exchange	2	0.8%	0.046^b^

a Pearson chi-square test

b Fisher’s exact test
